# Hunting for the elusive target antigen in gestational alloimmune liver disease (GALD)

**DOI:** 10.1371/journal.pone.0286432

**Published:** 2023-10-20

**Authors:** Klaus Rieneck, Karen Koefoed Rasmussen, Erwin M. Schoof, Frederik Banch Clausen, Henrietta Holze, Thomas Bergholt, Marianne Hørby Jørgensen, Vibeke Brix Christensen, Runar Almaas, Peter Lüttge Jordal, Marie Locard-Paulet, Kasper Runager, Leif Kofoed Nielsen, Balthasar Clemens Schlotmann, Joachim Lütken Weischenfeldt, Lars Juhl Jensen, Morten Hanefeld Dziegiel

**Affiliations:** 1 Laboratory of Blood Genetics, Department of Clinical Immunology, Rigshospitalet, Copenhagen, Denmark; 2 Department of Technology, Faculty of Health and Technology, University College Copenhagen, Copenhagen, Denmark; 3 Department of Biotechnology and Biomedicine, Technical University of Denmark, Kgs. Lyngby, Denmark; 4 Novo Nordisk Foundation Center for Protein Research, Copenhagen, Denmark; 5 Department of Obstetrics and Gynecology, Herlev Hospital, Herlev, Copenhagen, Denmark; 6 Department of Clinical Medicine, University of Copenhagen, Copenhagen, Denmark; 7 Department of Pediatric and Adolescent Medicine, Rigshospitalet, Copenhagen, Denmark; 8 Department of Pediatric Research, Oslo University Hospital, Oslo, Norway; 9 Institute of Clinical Medicine, University of Oslo, Oslo, Norway; 10 SBT Instruments A/S, Herlev, Denmark; 11 Teknologisk Institut, Aarhus C, Denmark; 12 Biotech Research & Innovation Centre, University of Copenhagen, Copenhagen, Denmark; Danmarks Tekniske Universitet, DENMARK

## Abstract

The prevailing concept is that gestational alloimmune liver disease (GALD) is caused by maternal antibodies targeting a currently unknown antigen on the liver of the fetus. This leads to deposition of complement on the fetal hepatocytes and death of the fetal hepatocytes and extensive liver injury. In many cases, the newborn dies. In subsequent pregnancies early treatment of the woman with intravenous immunoglobulin can be instituted, and the prognosis for the fetus will be excellent. Without treatment the prognosis can be severe. Crucial improvements of diagnosis require identification of the target antigen. For this identification, this work was based on two hypotheses: 1. The GALD antigen is exclusively expressed in the fetal liver during normal fetal life in all pregnancies; 2. The GALD antigen is an alloantigen expressed in the fetal liver with the woman being homozygous for the minor allele and the father being, most frequently, homozygous for the major allele. We used three different experimental approaches to identify the liver target antigen of maternal antibodies from women who had given birth to a baby with the clinical GALD diagnosis: 1. Immunoprecipitation of antigens from either a human liver cell line or human fetal livers by immunoprecipitation with maternal antibodies followed by mass spectrometry analysis of captured antigens; 2. Construction of a cDNA expression library from human fetal liver mRNA and screening about 1.3 million recombinants in *Escherichia coli* using antibodies from mothers of babies diagnosed with GALD; 3. Exome/genome sequencing of DNA from 26 presumably unrelated women who had previously given birth to a child with GALD with husband controls and supplementary HLA typing. In conclusion, using the three experimental approaches we did not identify the GALD target antigen and the exome/genome sequencing results did not support the hypothesis that the GALD antigen is an alloantigen, but the results do not yield basis for excluding that the antigen is exclusively expressed during fetal life., which is the hypothesis we favor.

## Introduction

Gestational alloimmune liver disease (GALD) is a disease that affects the liver of the fetus and newborn often with severe reduction of liver function [1–3]. It is the most prevalent form of neonatal hemochromatosis [1]. Untreated, the fetal mortality rate is high, but the woman is unaffected. The diagnosis of GALD rests on clinical and paraclinical observations whereas no specific diagnostic assay exists [4]. GALD is a rare disease with an incidence of approximately 4 per 100,000 births in the USA [5]. The pioneering work of Whitington and coworkers [6–8] indicated that the cause of the disease can be explained by transfer of maternal antibodies reactive against the fetal liver. Based on this, a rational treatment with intravenous immunoglobulin (IVIG) was instituted [8–10]. This treatment was very successful in subsequent pregnancies with close to 100% survival and little evidence of liver injury [8, 10, 11]. Animal experiments with injection into pregnant mice of IgG purified from GALD mothers have shown markedly reduced litter size or increased number of stillbirths [12, 13]. Liver histology from these mice showed extensive hepatocyte injury or necrosis. The outcomes of controls, injected with control sera, were similar to those from saline-injected animals [12, 13]. Liver histology from a narrow time window defined as E16 of P1 pups showed extensive hepatocyte injury or necrosis and increased lobular inflammation, while no other organs or tissues were damaged [12, 13]. Epidemiological data show that once a woman has given birth to a baby with GALD, there is approximately 80–95% risk that the subsequent babies will also suffer from GALD, even with another father [14–16]. Others have reported a recurrence rate of approximately 70% [17].

In some cases, accumulation of iron in the liver as well as extrahepatic siderosis is seen [1, 18, 19]. Extensive localization on fetal hepatocytes of terminal Complement complexes is a defining feature of GALD but not pathognomonic [1, 20, 21].

Taken together these observations suggest that maternal antibodies against one or more substance(s) expressed in or on the fetal liver are etiologically involved in GALD [1, 2]. Whether one or more antigen targets of antibodies from women with a child with GALD (GALD women [GW]) exist is not fully resolved at this time; also, the nature of the antigen(s) is unknown [1, 20]. Identification of the antigen targeted by maternal antibodies would expectedly enable the development of sensitive and specific diagnostic assays and thus through screening efforts help save the lives of otherwise healthy babies.

One hypothesis is that the maternal antibodies are directed against an antigen on the fetal liver exclusively expressed during fetal life in all normal pregnancies. In this case, probably several epitopes would be available as targets of maternal antibodies, and close to 100% of the fetuses of an immunized mother would be at risk without treatment.

An alternative hypothesis is that the antigen target is an alloantigen, i.e. an antigen that only some individuals in a population of the same species possess. The women are homozygous of a minor allelic variant and produce antibodies against the major paternally inherited allelic variant expressed in the liver of the fetus and adult liver tissue of those individuals. This situation is analogous to alloimmunization in many blood group systems. The recurrence rate of GALD in an untreated population of immunized women might directly reflect the frequency of the major allele in an alloimmune situation. There are no reports that first degree nor second-degree female relatives of a woman, who has had a child with GALD, have themselves had a child with GALD [8, 13, 22, 23]; in other words, there is no evidence of Mendelian inheritability of the GALD disease. However, this lack of evidence could be due to several factors: the production of a specific antibody is often associated with a specific HLA type that in the case of the alloantigen hypothesis need to be co-inherited with the putative minor GALD allele, and an immunizing event needs to take place. The low fecundity in families from western countries may also obscure a minor allele situation as causally involved in the rare GALD disease. Also, the rare diagnosis of GALD may likely be missed in some neonatal deaths.

**Fig 1:**
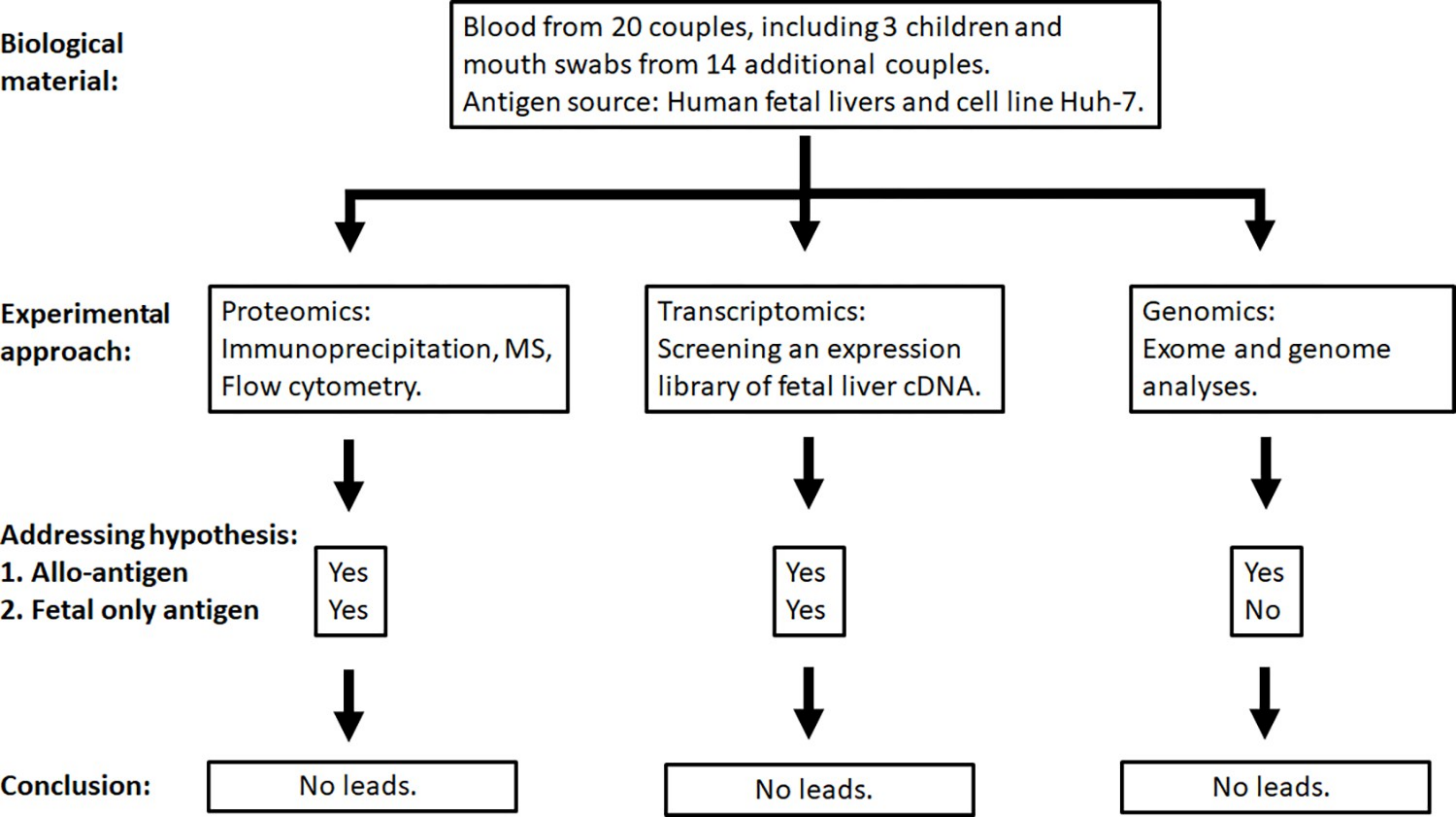
Overview of the three-pronged approach. A three-pronged approach was used to investigate the two hypotheses addressing the identity of the target antigen of GALD antibodies. Not all samples were investigated in all 3 experimental approaches. No convincing leads were identified from the 3 different experimental approaches.

We addressed both hypotheses in a three-pronged experimental approach (Fig 1):

1) Immunoprecipitation of antigens from an adult human liver cell line or fetal human liver from abortions at gestational age (GA) 19 and 21 by antisera from GW. The adult liver cell line reacted with antibodies from many GW in flow cytometry (FC) and was thus presumed to possibly express the GALD antigen; and

2) Construction and screening of an expression library in *E*. *coli*. The library was constructed from a pool of human fetal liver mRNA from different gestational ages and many recombinants were screened with antibody probes from GW

3) Analysis of genomes from 26 GW with the spouse genomes as controls and additionally 7 exomes from 3 couples and one child.

The current hypothesis of maternally produced antibodies as the cause of GALD is compatible with available epidemiological, clinical, and experimental evidence, but the aim to identify the GALD target antigen was not achieved. The current diagnostic basis of GALD is acute onset of liver failure, biochemical evidence of liver cell failure, coagulopathy persisting after vitamin K administration, increased iron in biopsies from submucosal glands indicative of extrahepatic siderosis, and biopsy of liver with staining for C5b-9 can be considered.

## Materials and methods

### Subjects

For the immunoprecipitation (IP) of antigen from human fetal livers, several GALD plasma and plasma from healthy controls were used in three experiments, see S1 Table for details.

For screening the expression library, plasma from two GW was used.

For the exome study, seven Danish subjects were included: one family (father and mother) with a newborn baby who died a few weeks after birth was included as well as the parents of two unrelated families who had several children and, in both families, at least one baby dead to GALD.

For the genome sequencing, the above mentioned seven subjects were included, and the rest of the adults were recruited internationally from countries belonging to the western hemisphere including Australia. Altogether 59 samples were obtained from GALD-affected families of which 50 samples were genome sequenced. 26 samples were from the women, 22 were from the men, and 2 were children. 19 samples were mouth swaps (9 women and 10 men), 31 were whole blood samples (2 children, 17 women and 12 men).

**Fig 2:**
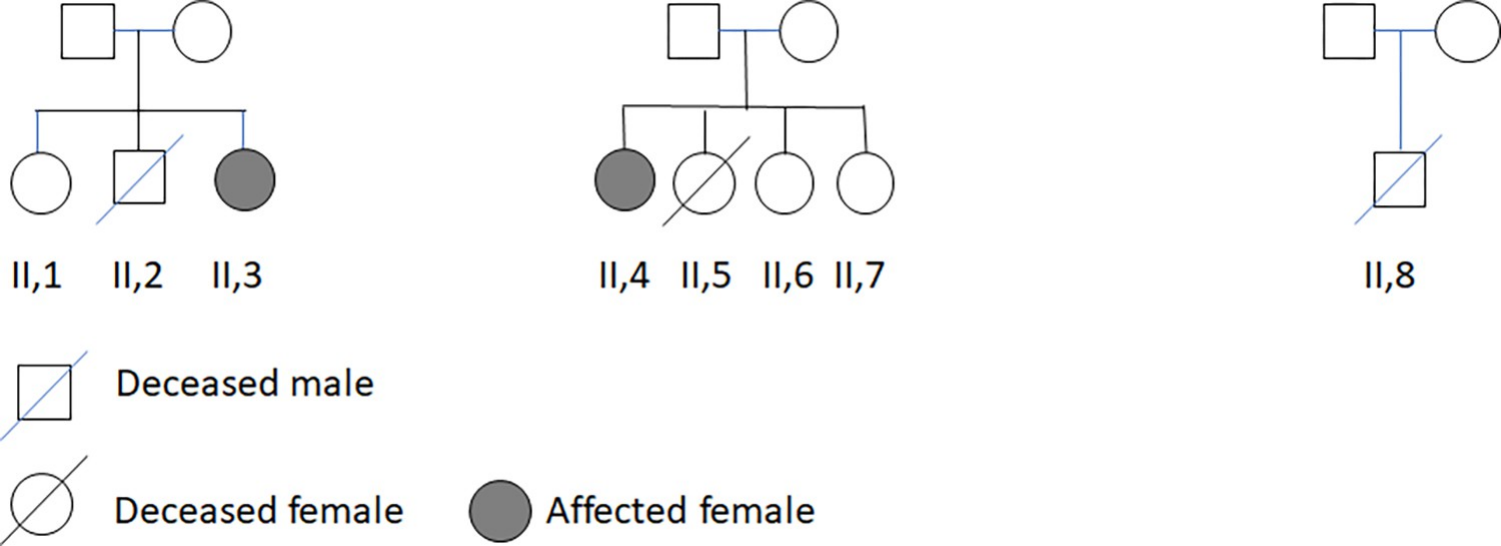
Exome analysis pedigree. The pedigrees of the three families included in this study for the exome analysis are shown. DNA from the fathers and mothers was used in the exome analysis as well as DNA from the child II.8. For children II.6 and II.7 the mother was treated with IVIG during pregnancy. The diagnosis of GALD was clinically verified for the affected and deceased children.

The diagnosis of GALD was established by clinical, paraclinical, and pathological criteria. The Danish families and the newborns are shown in pedigrees in Fig 2.

All the women included in this study, except for the controls, had given birth to at least one baby diagnosed with GALD.

### Plasma

Plasma from all the subjects who donated whole blood was produced by centrifuging EDTA-anticoagulated blood at 3100xg for 10 min at room temperature (RT). Plasma was pipetted into sterile polypropylene tubes and frozen at -20°C and stored at -80°C.

Control plasma for the IP and FC analysis was anonymized leftover plasma from blood donors, but blood from those donors who had indicated that they did not want use of their blood for research or quality purposes was not used as controls. Plasma from a woman, who has given birth to a baby diagnosed with GALD, will in the following be referred to as GALD plasma.

### Cell lines

The human liver cell line Huh-7 (JCRB Cell Bank [Japanese Collection of Research Bioresources Cell Bank] cat no JCRB0403) was derived from a liver tumor from an adult Japanese male in 1982 and was described as a carcinoma. The Huh-7 cell line was cultivated in a 37°C humidified incubator with 5% CO_2_. The cells were grown to 70–80% confluency in T175 cell culture flasks (NUNC, Copenhagen, Denmark) in Advanced DMEM (Gibco, Taastrup, Denmark) supplemented with 1% Glutamax (Gibco), penicillin/streptomycin (Gibco) to a final concentration of 50 μg/mL, and 10% Fetal Bovine Serum (FBS) (Gibco).

The medium was changed every second day, and cells were seeded at densities of approximately 4-5x10^5^ viable cells/mL. When the cells reached a confluency of about 70–90% they were re-plated by loosening with Tryple (Gibco) for about 5 min at RT. The cells were collected by centrifugation and washed once with PBS without Ca^2+^ or Mg^2+^. Cells were stored long-term in 10% DMSO (Sigma-Aldrich, Brøndby, Denmark) and 40% FBS at densities of about 5x10^6^ cells/mL in liquid nitrogen. The cells were thawed according to standard protocols.

### Fetal human livers

From the HDBR tissue bank (Human Developmental Biology Resource, HDBR.org) we obtained human fetal livers or fragments of livers from GA 19 and 21.

### Bacteria

XL1-Blue MRF’ were kept as stocks at -80°C in 15% glycerol stocks. For plaque screening, the bacteria were grown overnight in 25 mL Luria Broth (LB) with tetracycline at 12,5 ug/mL under shaking in an orbital incubator. The next day, 500 μL bacteria were transferred into a new 25 mL LB without antibiotics but including 500 μL 20% maltose. After 3 hours incubation at 37°C in the orbital incubator at 250 rpm, the cells were pelleted at 1700xg for 10 min, the supernatant was discarded, and the bacterial pellet resuspended in 7 mL 10 mM MgSO_4_. OD600 was checked and was adjusted to be close to 1. These bacteria were used within two days.

### HLA determination

16 of the GW were high-resolution HLA typed by genetic methods for both class I and class II antigens using third-generation DNA sequencing (Histogenetics, NY, USA). The HLA typing of the women was done to establish if certain HLA types would be overrepresented.

### Immunoprecipitation and mass spectrometry (MS) of human liver cell line Huh-7 and human fetal liver tissue

We used two antigen sources, the hepatocyte cell line Huh-7 and frozen fetal liver tissue from GA 19 and 21 respectively. Two protocols were used: one was aimed at investigating the Huh-7 cell line and the other at investigating human fetal liver samples. Huh-7 cells were first shown to react with plasma from GW above control plasma, justifying the use of this cell line derived from an adult as the antigen source [24]. An overview of the experimental design is shown in S2 and S3 Tables.

#### Huh-7: IP of antigens from the Huh-7 hepatocyte carcinoma cell line

For IP with Huh-7, plasma from EDTA anti-coagulated whole blood from GW was used in the study. Positive technical control was EDTA plasma from a woman suffering from primary biliary cirrhosis (PBC). Negative control was EDTA plasma from normal female blood donors. Huh-7 cells were lysed by vigorous pipetting with 1X RIPA buffer (Abcam) with protease inhibitor (Protease Inhibitor Cocktail (PIC), Abcam). Lysate was incubated at RT for 1 hour on an end-over-end mixer, followed by centrifugation at 4°C at 16,000xg for 20 minutes, and the supernatant designated Huh-7 lysate was harvested. The concentration of protein was 4.7 mg/mL as measured by Qubit. First, 100μL protein G beads suspension (Thermo Fisher) was placed in 1.5mL low adsorbance Eppendorf microtubes in a DynaMag™- Spin Magnet (Thermo Fisher), drained and incubated with 100μL plasmafrom EDTA anti-coagulated whole blood. Before incubation with beads, plasma was diluted with 100μL PBS+1% Tween20 (PBS-T). Beads and plasma were incubated for 1.5 hours at RT to ensure complete loading of IgG on beads. Beads were then consecutively washed 3x with 500μL PBS-T, 2x with 500μL RIPA buffer, 3x with 500μL PBS-T, and were finally resuspended in 100μL PBS-T. 75μL-beads suspension was incubated overnight with 300μL Huh-7 lysate end-over-end at 4°C. The remaining 25μL were stored at 4°C for later use.

Beads were washed on the magnet 4x with 500μL RIPA buffer, 4x with 500μL PBS-T, and finally resuspended in 75μL PBS-T. Beads were transferred to new tubes for each washing to avoid carry over from tube surfaces. Antigens on beads were eluted with 25μL 31mM citrate buffer (25 mM KH2PO4, 56 mM NaCl pH 2.31) for 5 minutes, the supernatant was removed from beads and neutralized with 0.1 volume 1M PBS pH 7.5. Elution was repeated. The first and second eluates were pooled and stored at -20°C before further processing.

#### Huh-7: MS sample preparation of antigens from Huh-7 cells

Relative protein quantification was determined using the iTRAQ 8-plex (iTRAQ®, AB Sciex) that allows relative comparison of 8 samples. Protein concentration of samples was determined using Pierce BCA Protein Assay Kit following manufacturer’s protocol. Proteins from each sample were acetone-precipitated overnight. Pellets were dissolved in 20μL Dissolution Buffer with 1 μL Denaturant (iTRAQ^®^ Reagents, AB Sciex). Proteins were reduced and subsequently alkylated following manufacturers protocol and using reagents from the iTRAQ assay (iTRAQ^®^ Reagents, AB Sciex). Proteins were enzymatically digested overnight to peptides using a 1:50 trypsin:protein ratio (Sequencing Grade Modified Trypsin, Promega Biotech AB).

Each sample was labeled with isobaric tags to allow relative quantitation (iTRAQ). Peptides were incubated with the specific iTRAQ labeling reagents at RT for two hours. Hereafter, all eight samples were pooled to an 8-plex for subsequent strong cation exchange (SCX) fractionation. Prior to offline SCX fractionation, sample volume was reduced to <50μL by vacuum centrifugation. Loading buffer (10% formic acid) was added for acidification of sample. SCX fractionation was performed using HyperSep SCX SPE columns (Thermo Scientific) and Visiprep SPE Vacuum manifold (Sigma-Aldrich). Columns were conditioned by 1mL 100% ACN, 500μL pH 4 SCX elution buffer, 500μL pH 8.5 SCX elution buffer, 1mL 0.1% Trifluororacetic acid (TFA) and 40% acetonitrile (ACN). Acidified samples were loaded on columns and fractions were subsequently eluted by increasing pH. SCX elution buffers were 20 mM acetic acid, 20 mM boric acid, 20 mM phosphoric acid and 50% ACN, adjusted to 10 pH levels (pH 4.0, 4.5, 5.0, 5.5, 6.0, 6.5, 7.0, 7.5, 8.5, 11.0) by 5M NaOH. Fractions 4.0+4.5 and 8.5+11.0 were pooled to attain eight fractions for further purification and analysis. Volume of SCX fractions of each sample were reduced to <100μL by vacuum centrifugation and fractions were acidified by 10% FA. All sample fractions were then purified by Sep-Pak^®^ Vac C18 cartridges (Waters) on a Visiprep SPE Vacuum manifold (Sigma-Aldrich) prior to nLCMS/MS analysis.

#### Huh-7: MS data acquisition

The purified acidified 8-plex was analyzed by high-resolution electrospray ionization tandem mass spectrometry (ESI-MS/MS). Reversed phase nanoLC separation (Dionex UltiMate RSLCnano System) was performed online coupled to the mass spectrometer (LTQ Orbitrap Velos). Peptides were loaded on the column (Prep C18 StageTip) using 96% buffer A (0.1% Formic acid in water, LC-MS grade) and 4% buffer B (0.1% Formic acid, 80% Acetonitrile in water, LC-MS grade). Peptides were eluted by a 1.5–3 hour gradient of increasing proportion of buffer B. The 8-plex was analyzed using eight subsequent injections. Tandem mass spectrometry parameters were: positive mode, MS scan range 300–1600 m/z with resolution at 30,000, MS/MS fragmentation was performed using HCD (higher-energy collisional dissociation) on the 15 most intense ions with a normalized collision energy of 40, dynamic exclusion of 90 s and a minimum signal threshold of 10,000.

#### Huh-7: Protein identification and quantification

Raw MS files were analyzed by MaxQuant version 1.6.17.0 [25]. Data were searched with the Andromeda search engine against the human reference proteome from UniProt (downloaded on 11.11.2020) containing 75069 protein sequences from UniProtKB and a list of potential contaminant sequences provided in MaxQuant 1.6.17.0. Specificity of trypsin digestion was set for cleavage after lysine or arginine, and two missed cleavages were allowed. Precursor tolerance was set to 20 ppm and 4.5 ppm for the first and main search, respectively. The MS/MS tolerance was set to 20ppm for Fourier Transform (FTMS) and 0.5 Da for Ion Trap (ITMS). Validation was performed through a false discovery rate (FDR) set to 1% at protein and peptide-spectrum match (PSM) level determined by target-decoy search in MaxQuant. The minimum peptide length was set to 7. Proteins were considered identified with minimum 1 razor peptide and protein quantification were based on unique and razor peptides.

For analysis of IPs with Huh-7 cell line, the search included methionine oxidation, protein N-terminal acetylation and Deamidation (N) as variable modifications and as fixed modification Methylthio on the cysteines (C) (Unimod accession 39). Reporter ion purity correction was performed based on correction factors communicated by the provider.

#### Huh-7: Data analysis

We analyzed the relative quantities of protein groups identified by MaxQuant in the 8 raw files. 3 samples with GALD plasma were compared against 4 samples with healthy plasma and as a positive control one sample with PBC plasma against samples with healthy plasma. The quantitative proteomic analysis was performed using the statistical package R v4.1.1 (R Core Team, 2021; http://www.R-project.org/).

Proteins identified with no unique peptides were removed from the analysis. The corrected intensity of the reporter ion from the “proteinGroups.txt” table of MaxQuant was log_2_ transformed and normalized with quantile normalization. Principal component analysis (PCA) was performed to identify the main sources of variation in the data and the effect of the normalization.

Linear Models For Microarray Data (LIMMA) as implemented in the limma R package v3.48.3 was used to identify differentially abundant proteins between GALD samples and healthy controls [26]. Fold changes and standard errors were estimated by fitting a linear model to each protein via the lmFit function and applying empirical Bayes smoothing via the eBayes function. False discovery rates (FDRs) were calculated using the Benjamini-Hochberg procedure [27] and proteins with FDR < 0.05 were considered as significantly differentially abundant.

Code for data analysis and figures is available at https://github.com/HenriettaHolze/GALD_proteomics.

#### Fetal liver tissue: IP of antigens from human fetal liver tissue

Approximately 600 mg frozen human fetal liver tissue (from GA 19 and 21) was divided into four 2mL vials and placed on dry ice for 15 min. A pre-cooled 5mm steel ball was added to each vial, and the tubes were placed in the Tissuelyzer Insert (Qiagen). To prevent that the following addition of RIPA buffer would result in freezing of the buffer, the vials were left at RT for 2 min. 700 μL cold RIPA buffer (Abcam) with protease inhibitor solubilized in DMSO to a final concentration of 1X (Protease Inhibitor Cocktail, Abcam) was added, and the tissue was homogenized with a TissueLyzer LT (Qiagen) for 3 minutes at 50 Hz at RT. The lysates were pooled in one 50mL NUNC tube (Nunc, Roskilde, Denmark) and sonicated (3 x 10 sec pausing on ice in-between). The sample was transferred to a centrifuge tube and was centrifuged at 16,000x*g* for 20min at 4°C and the supernatant was harvested in a 50 mL NUNC tube and stored on ice until further use.

For IP with fetal liver, 0.22μm sterile-filtered plasma from seven different GW (positive samples), four normal female donors (negative controls) and one female patient with PBC (positive technical control) was used. In the following section, low protein bind Eppendorf tubes were used in all steps. For each sample, 100 μL Protein G bead suspension (Immunoprecipitation Kit Dynabeads Protein G (Thermo Fisher Scientific)) was transferred to each Eppendorf tube and placed on a magnet to allow draining. Subsequently 50 μL GW or control plasma was added to each tube and left for incubation for 4 hours at 4°C on an end-over-end mixer according to the manufacturer’s instructions. Beads were washed once in 1 mL RIPA buffer and subsequently 3x in 1 mL PBS-T. 400 μL human fetal liver tissue lysate was added to each vial and incubated end-over-end overnight at 4°C. Beads were washed once with 1 mL RIPA buffer, 2x in 1 mL cold PBST, and 3x in 1 mL cold PBS pH 7.4. After the last wash the beads were drained and stored at -80°C until further analysis.

#### Fetal liver tissue: MS sample preparation of antigens from human fetal liver tissue

The beads were resuspended in 20 μL of lysis buffer (consisting of 6 M Guanidinium Hydrochloride, 10 mM Tris Carboxy Ethyl Phosphene (TCEP), 40 mM chloroacetamide (CAA), 100 mM Tris pH 8.5). Samples were boiled at 95°C for 5 minutes, after which they were sonicated on ‘high’ intensity for 3x 10 seconds in a Bioruptor sonication water bath (Diagenode) at 4°C. Samples were then taken forward for digestion, and diluted 1:3 with 10% Acetonitrile, 25 mM Tris pH 8.5. 100 ng of LysC (MS grade, Wako) was added, and samples were incubated at 37°C for 4 hours. Samples were further diluted to 1:10 with 10% Acetonitrile, 25 mM Tris pH 8.5, and 100ng of trypsin (MS grade, Sigma) was added and samples were incubated overnight at 37°C. Enzyme activity was quenched by adding 2% trifluoroacetic acid (TFA) to a final concentration of 1%. Prior to mass spectrometry analysis, the peptides were desalted on in-house packed C18 Stagetips [28]. For each sample, 2 discs of C18 material (3M Empore) were packed in a 200μL tip, and the C18 material activated with 40μL of 100% Methanol (HPLC grade, Sigma), and then 40μL of 80% Acetonitrile, 0.1% formic acid. The tips were subsequently equilibrated 2x with 40μL of 1%TFA, 3% Acetonitrile, after which the samples were loaded using centrifugation at 3050xg. After washing the tips twice with 100μL of 0.1% formic acid, the peptides were eluted into new 500μL Eppendorf tubes using 40% Acetonitrile, 0.1% formic acid. The eluted peptides were concentrated in an Eppendorf Speedvac, and re-constituted in 1% TFA, 2% Acetonitrile for MS analysis.

#### Fetal liver tissue: MS data acquisition

For each sample, peptides were loaded onto a 2 cm C18 trap column (ThermoFisher 164705), connected in-line to a 50cm C18 reverse-phase analytical column (Thermo EasySpray ES803) using 100% Buffer A (0.1% Formic acid in water) at 750bar, using the Thermo EasyLC 1200 HPLC system, and the column oven operating at 45°C. Peptides were eluted over a 140 minute gradient ranging from 6 to 60% of 80% acetonitrile, 0.1% formic acid at 250 nL/min, and the Orbitrap Fusion instrument (Thermo Fisher Scientific) was run in a DD-MS2 top speed method. Full MS spectra were collected at a resolution of 120,000, with an automatic gain control (AGC) target of 4×10^5^ or maximum injection time of 50 ms and a scan range of 400–1500 m/z. The MS2 spectra were obtained in the ion trap operating at rapid speed, with an AGC target value of 1×10^4^ or maximum injection time of 35 ms, a normalized HCD collision energy of 30 and an intensity threshold of 1.7e^4^. Dynamic exclusion was set to 60 s, and ions with a charge state <2, >7 or unknown were excluded. MS performance was verified for consistency by running complex cell lysate quality control standards, and chromatography was monitored to check for reproducibility.

#### Fetal liver tissue: Protein identification and quantification

Raw files were analyzed the same way as for IPs with Huh-7 except for the following changes. The search included methionine oxidation and protein N-terminal acetylation as variable modifications and carbamidomethylation of cysteines as a fixed modification. The “match between runs” option of MaxQuant was enabled for label-free relative quantification of peptide ions across runs [25]. The minimal ratio count was set to 2 for calculation of label free quantification (LFQ) intensities.

**Fetal liver tissue: Data analysis.** The IPs were analyzed separately due to differences in antigen source and preparation methods and samples were excluded from the statistical analysis if they contained no plasma, plasma from patients under treatment, or were prepared differently than control samples (pre-incubated samples in IP1).

Proteins that were differentially abundant in samples with GALD and healthy plasma were identified based on the (LFQ) intensities from the “proteinGroups.txt” table of MaxQuant. Proteins identified with no unique peptides were removed from the analysis. Log_2_ transformed LFQ intensities were realigned to the mean median signal.

LIMMA analysis was performed for each IP separately the same way as for Huh-7 samples. LIMMA was only applied to protein groups present in both control and GALD samples. The protein groups only quantified in one condition but at least 2 samples were plotted with an average fold change calculated against the lower 0.1 percentile of the respective IP.

Code for data analysis and figures is available at https://github.com/HenriettaHolze/GALD_proteomics.

### Expression library

#### Construction of cDNA expression library

Construction of a cDNA expression library was done in the bacteriophage lambda ZAP vector (Agilent, Glostrup, Denmark). The recommendations of the manufacturers were followed with a few modifications. Briefly, a cDNA library in the lambda ZAP expression vector was constructed from commercially obtained mRNA from human fetal livers from 38 spontaneously aborted fetuses of GA ranging from 22–40 (Clontech, Herlev, Denmark). The library was produced by ligating lambda ZAP EcoRi/CIAP treated vector arms to insert produced from oligo dT primed mRNA and EcoRi adaptors appended to the double-stranded cDNA insert. The adaptors were hemiphosphorylated during synthesis (Eurofins), the adaptor-ligated inserts were fractionated on a Sephacryl S-500 minicolumn (Invitrogen). 50–100 ng cDNA insert was ligated to 1 μg EcoRi digested and CIP treated lambda vector arms overnight at 12°C and 1–3μL ligation mix was packaged with Gigapack III Gold packaging kit (Agilent) according to the manufacturer’s instructions.

#### Screening of expression library

LB medium, top agar, and LB plates were made by a local service provider (Department of Substrates, Panum, University of Copenhagen), IPTG, and X-gal (Sigma-Aldrich) were used for the testing of the expression library.

For screening of the expression library, 5x10^4^ plaque forming units were mixed with 600 μl fresh XL1-blue prepared as described, incubated at 37°C for 15 min. Then 7.5 mL top agar, molten and temperature equilibrated at 48°C was added to the bacteriophage/XL1-blue and briefly vortexed and quickly spread on a 150 mm diameter agar plate, and left at RT for about 10 min. The plates were incubated at 37°C for 4 hours. Plaque lifts for screening the expression library with antibody probes were done with BA85 nitrocellulose membrane (Whatman, GE Healthcare, Knebel, Denmark) 132 mm in diameter. The membranes were wetted by and submerged in 10 mM IPTG and briefly airdried on a Whatman 3MM blotting paper. The membranes were then carefully placed on the top agar avoiding air bubbles and incubation was continued at 37°C for 3.5 hours. After this incubation, the plates were transferred to 4°C overnight. The position of the membranes on the top agar was marked by punching through the membrane while on the agar plate with a 16G needle and the membranes were subsequently carefully lifted without removing any top agar. Membranes were washed in a large volume (>70 mL pr membrane) of TBST (20 mM Tris pH 7.5, 150 mM NaCl 0,05% Tween 20) at RT for 15 min. The membranes were blocked in 1% BSA in 0.1 M Tris, pH 7.4, 1 M NaCl, 0.005% Tween 20 (Merck).

The expression library was screened with antiserum obtained from GW. The patient antiserum was first absorbed for anti-*E*. *coli* reactivity by incubating 10 μL anti-serum in 10 mL blocking buffer with about three grams of *E*. *coli* from an overnight culture and inverting the tube gently on a blood mixer for 1 hour at RT. The supernatant was used after centrifugation at 2000x g for 10 min. The pellet was discarded. 10 mL was used for a membrane of 82 mm in diameter and 30 mL for membranes of 132 mm diameter. After incubation for 1 hour at room temperature with the primary antibody, membranes were washed 3 times at room temperature with TBST as described above.

The secondary antibody P0214 (Agilent), a murine monoclonal anti-human IgG antibody conjugated to horse radish peroxidase was used in a dilution of 1:1000 in TBST, and membranes were washed 3 times at room temperature with TBST as described above and were developed in TMB D blotting solution (Kem-En-Tec, Taastrup, Denmark) for approximately 10 min at RT.

About 1,000,000 plaques were screened with *E coli* absorbed patient plasma, and about 300,000 additional plaques from an unamplified library were screened with patient plasma absorbed and eluted from Huh-7 cells. Huh-7 cells were cultivated in multilayer plates under conditions described, and a total of 10^9^ cells were obtained after detaching the cells with Tryple and washing two times with PBS. It was previously shown by FC with GW plasma that Huh-7 cells scraped of the flasks gave slightly less binding with plasma from GW than Tryple digested, fixed Huh-7 cells. The Huh-7 cells were treated with fixability dye FVD450 (Invitrogen) and 10 μl cells were fixed with 1 mL 2% glutaraldehyde (Sigma Aldrich) for 30 min at RT. The cells were then washed 3 times with PBS. Plasma from GW was added to fixed Huh-7 cells and incubated for 30 min RT. The cells were washed 3 times with PBS and the bound antibody eluted with 1 volume of citric acid buffer pH 2.8 (62mM citric acid, 48 mM NaH_2_PO_4_, 154mM NaCl) for 2 min, the cells were immediately centrifuged for 2 min, and the supernatant was neutralized with 0.25 times the volume of sodium phosphate buffer pH 12.8 (Ampliqon, Odense, Denmark). The antibodies eluted from the Huh-7 cells were checked on flowcytometry to bind to Huh-7 cells and was used for screening the recombinant phages. The eluted antibodies gave less back-ground than the antibodies that were *E*. *coli* adsorbed and a few weakly positive plaques were picked and eluted in SM buffer [29] and the phages were re-screened using the same procedure.

### DNA sequencing

#### Exome sequencing

The three Danish couples and one infant were exome sequenced at 100x coverage. Briefly, the library was constructed using an Agilent SureSelectXT Human AllExon 60 Mb enrichment kit and NuGEN library preparation. The sequencing was done on a NextSeq 500 v2 instrument (Illumina, CA, USA) with 2x150 bp reads. Alignment of quality trimmed reads against the reference genome hg19 was done using, Burrows–Wheeler alignment—MEM, maximal exact match.

BWA-MEM v 0.7.12. Variant discovery of samples was done with FreeBayes v1.0.2. IGV software was used for viewing BAM and VCF files. Second-level analysis was done by Ingenuity software (Ingenuity, Qiagen, Copenhagen) after uploading of VCF files. The analysis was done as trio-analysis and as two-group analysis and a combination of analysis of the individual couples searching for homozygotic variants, compound heterozygotic variants, and de novo mutations. The question was if the women predictably would not express an antigen that the fathers would pass on to the child allowing for maternal antibodies to target such a hypothetical alloantigen on fetal liver cells.

#### Genome sequencing

Whole-genome sequencing was performed by deCODE genetics, which has been previously described in detail [30, 31]. In short, paired-end libraries for sequencing were prepared from isolated DNA samples using Illumina preparation kits (TruSeq DNA, TruSeq Nano, or TruSeq PCR-Free) according to the manufacturer’s instructions. Paired-end sequencing-by-synthesis was performed on Illumina sequencers (GAIIx, HiSeq 2000/2500, HiSeq X, or NovaSeq) to a target depth of 30×. Reads were aligned to the human genome assembly GRCh38 using the Burrows–Wheeler Aligner version 0.7.10 [32]. Alignments were merged into a single BAM file and marked for duplicates using Picard 1.117. Variants were called using version 2014.4-2-g9ad6aa8 of the Genome Analysis Toolkit (GATK) [33]. The effects of sequence variants were annotated using release 80 of the Variant Effect Predictor (VEP-Ensembl) [34].

#### Additional genome analysis

Ancestry and relatedness analysis was done with Somalier 0.2.11 [35].

### Ethics

The project conformed to the Helsinki II declaration and was approved by the local ethics committee under protocol numbers H-16042284 and H-15014312 and several supplementary add-on protocols. Participants gave written informed consent, and for the participating children, their parents gave written informed consent.

The recombinant DNA part of the project was duly reported to The Danish work environment authority (Arbejdstilsynet), and the project was reported to the system of the data overseeing authority (Pactius) and approved under application number P-2020-1118.

## Results

### Mass spectrometry of immunoprecipitated antigens

**Fig 3:**
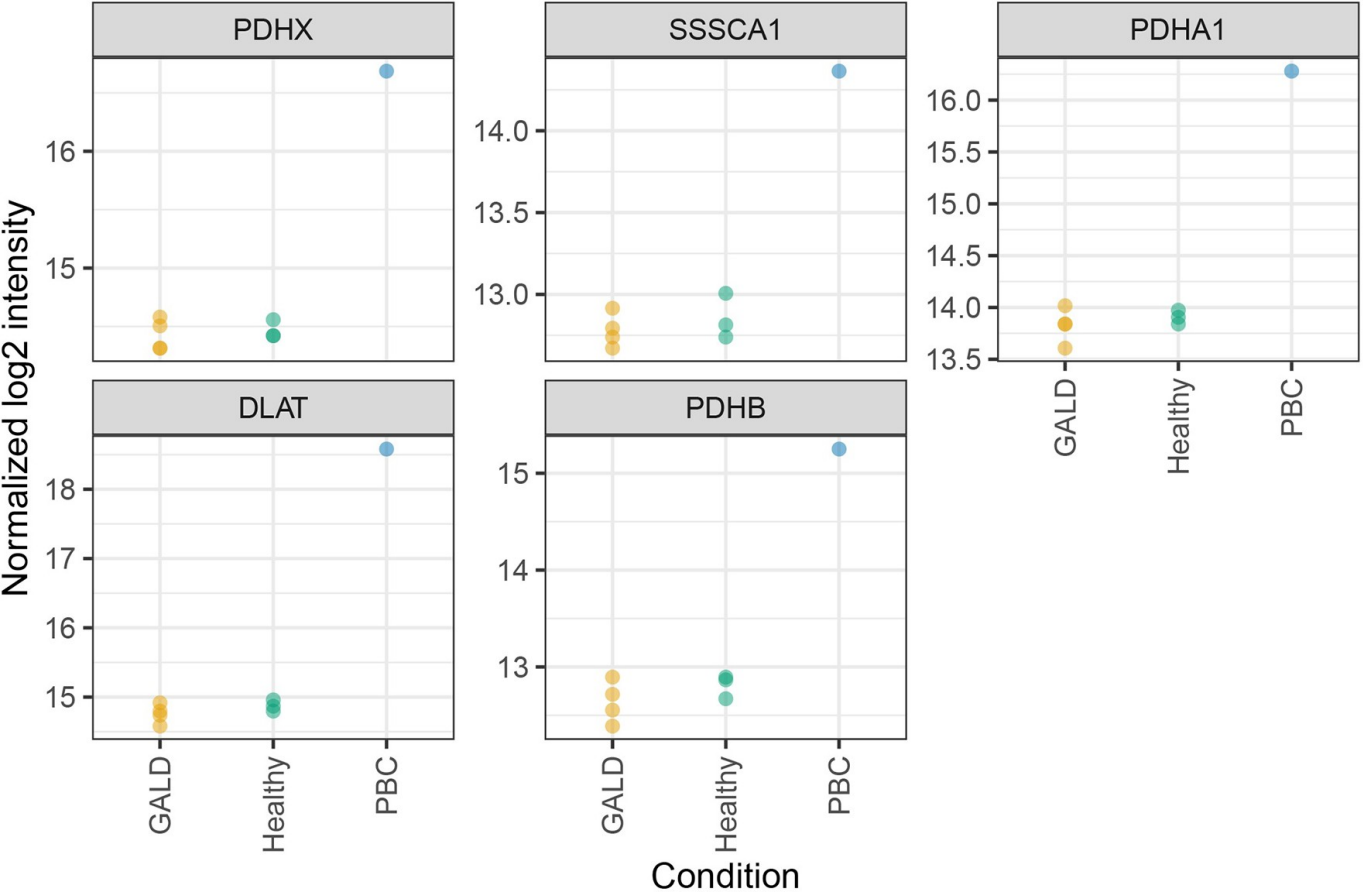
Immunoprecipitaion from Huh-7. Normalized log_2_ intensities of Primary Biliary Cirrosis (PBC) antigens in IPs with Huh-7 cell line.

The IPs were performed using GALD plasma, normal control plasma and plasma from a patient with primary biliary cirrhosis (PBC). The mitochondrial pyruvate dehydrogenase complex is a known antibody target in PBC [36]. To ensure the efficacy of the IPs of antigens, known antigen-targets in PBC were checked in the data (S4 Table). Plasma from a PBC patient thus served as control of antigen IP. Five known antigen targets were precipitated more abundantly by the PBC plasma sample compared to samples with healthy and GALD plasma in immunoprecipitation using Huh-7 as antigen donor material (Fig 3).

**Fig 4:**
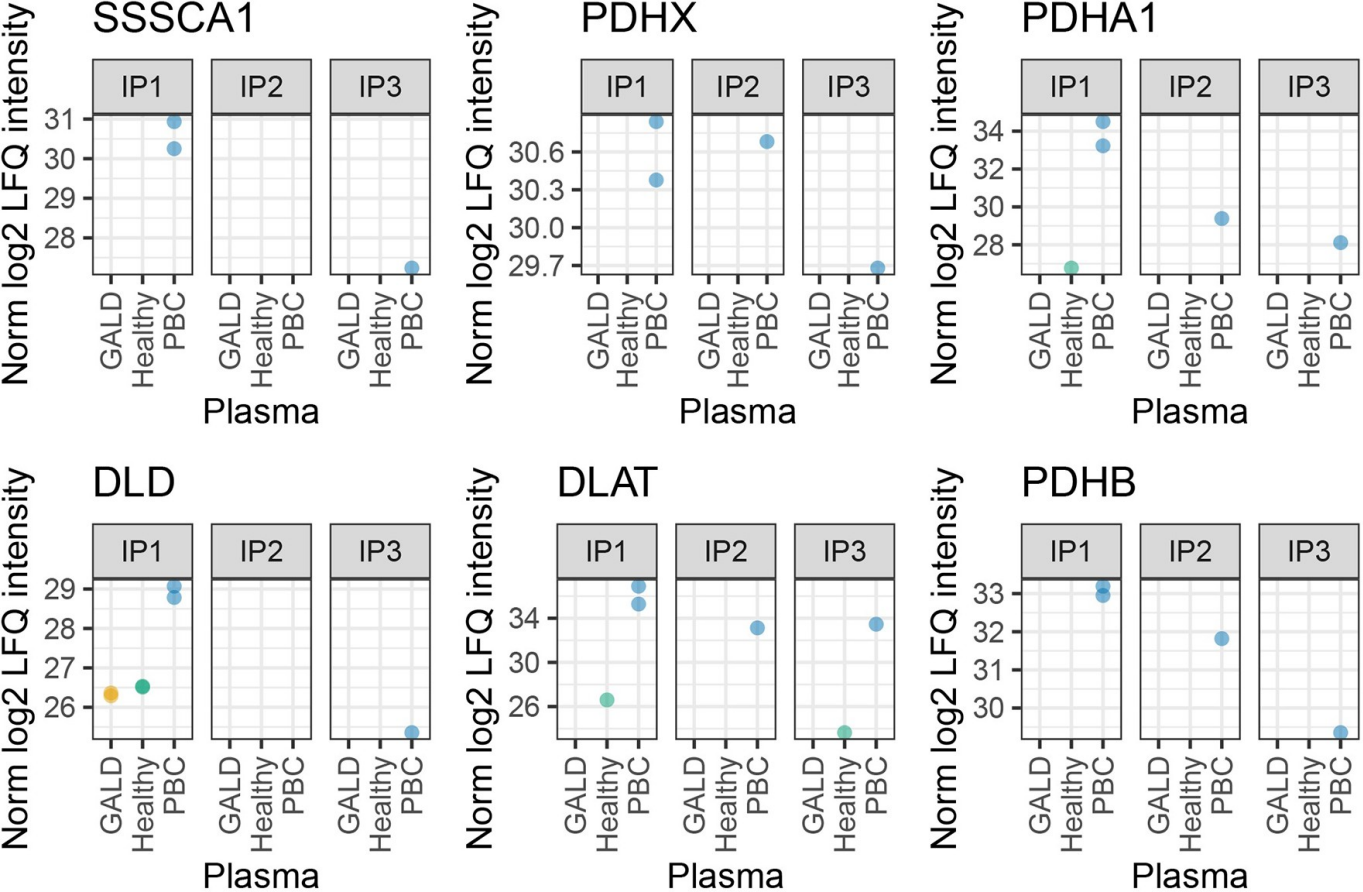
Immunoprecipitation from human fetal liver. Normalized log_2_ Label Free Quantification (LFQ) intensities of PBC antigens in IPs with fetal liver material.

Six PBC antigens were either only quantified or more abundant in samples with PBC plasma compared to healthy controls when using human fetal liver as antigen donor material (Fig 4).

**Fig 5:**
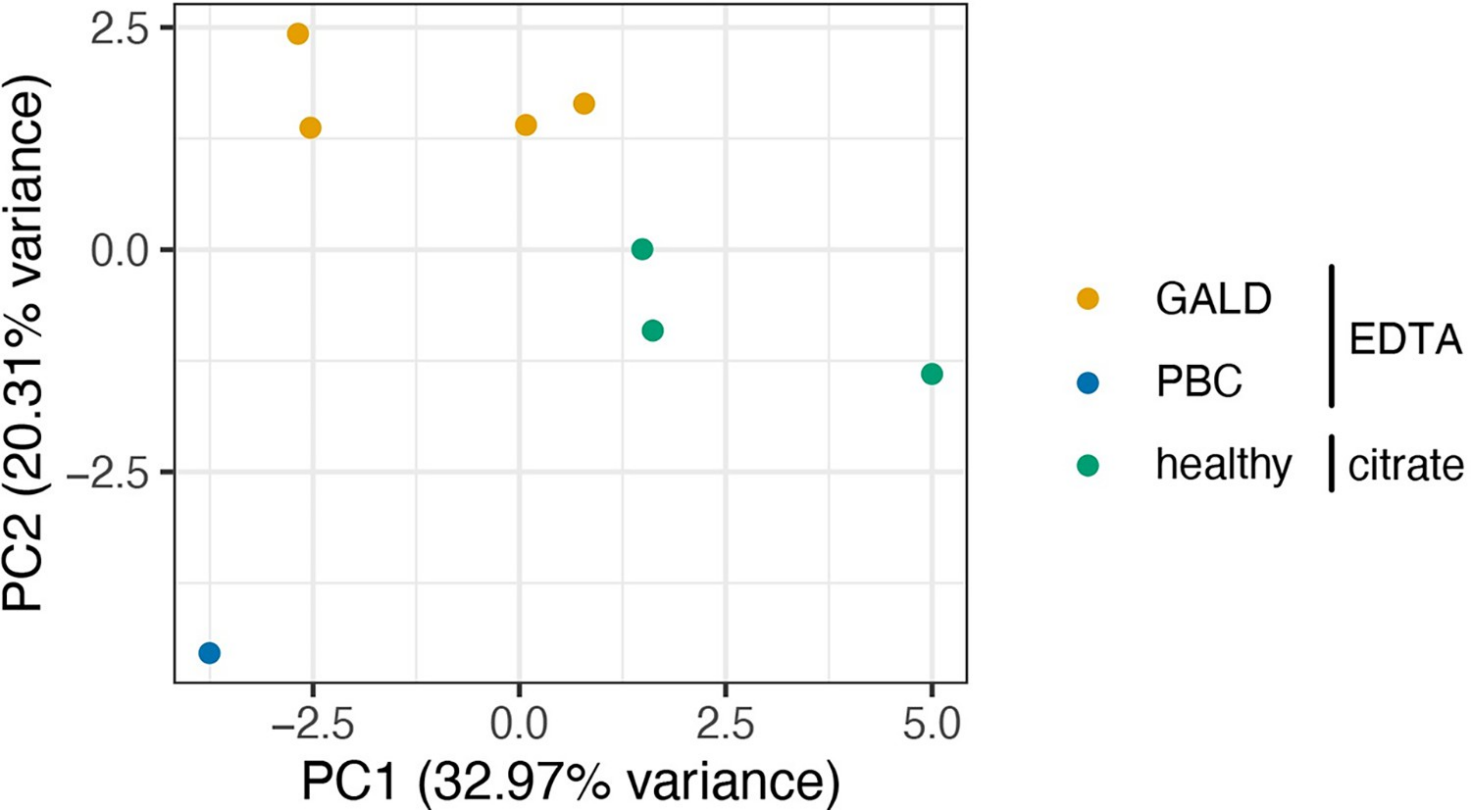
Principle components 1 and 2 of PCA of MS results of IPs with Huh-7 cell line. The PBC sample and the GALD samples were prepared with EDTA while the healthy samples were prepared with citrate.

212 protein groups were quantified in the samples, and PCA showed that the samples could be separated by condition on principal components 1 and 2 (Fig 5).

**Fig 6:**
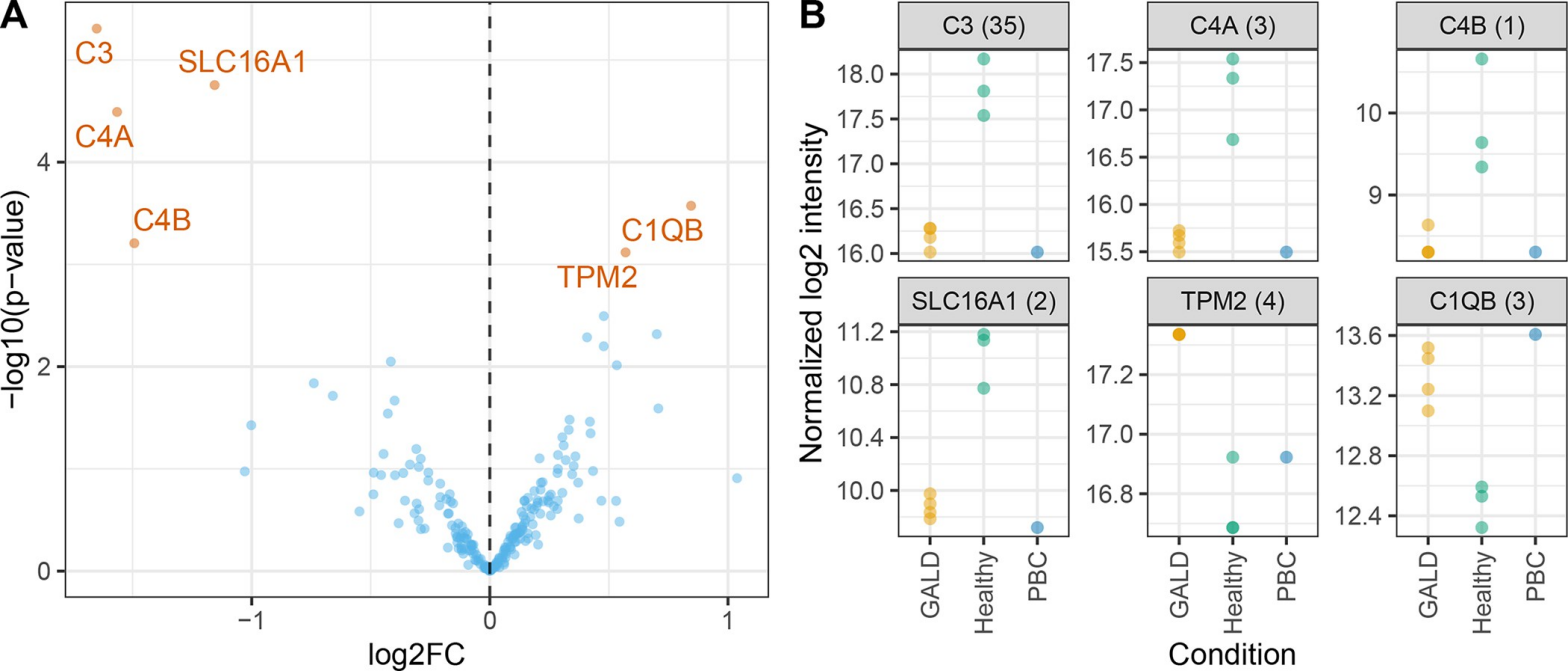
Differential protein abundance in GALD plasma vs healthy controls in Huh-7 cell line. A) Volcano plot depicting differential abundance of proteins in IPs with GALD plasma vs healthy plasma with Huh-7 cell line. Proteins with Benjamin-Hochberg adjusted p-value (FDR) < 0.05 in orange: Complement C3 (C3), Complement C4-A (C4A), Complement C4-B (C4B), Monocarboxylate transporter 1 (SLC16A1), Tropomyosin beta chain (TPM2), Complement C1q subcomponent subunit B (C1QB). B) Normalized log_2_ LFQ intensities of significantly differentially abundant proteins in 3 conditions GALD, healthy and PBC plasma. Number of unique peptides in brackets. LogFC = log Fold Change.

To identify potential antigens that were bound by antibodies from the plasma of GW, we identified proteins that were differentially abundant IP in samples with GALD plasma *versus* samples with healthy plasma using LIMMA. Six proteins were significantly differentially abundant (FDR < 0.05, Fig 6, S5 Table). Two proteins (TPM2, C1QB) were identified to be significantly more abundant in IP samples of GALD plasma than in plasma from healthy controls, which would make them potential target antigens of maternal antibodies for development of GALD.

It was later realized that a different anti-coagulant was used for healthy control plasma. PBC and GALD plasma were taken in EDTA tubes whereas citrate tubes were used for the control samples. PC1 of the PCA separated the samples into diseased (GALD, PBC) and healthy plasma but also into EDTA and citrate samples (Fig 5). The difference in anti-coagulant was most likely the reason for differential abundance of e.g. C1QB. Thus, no plausible candidate antigens were identified by IP and MS analysis of molecules from the Huh-7 cell line.

Three IPs (S3 Table) were then performed with fetal liver material as antigen source and healthy, GALD and PBC plasma. The number of proteins identified in each IP after QC (# proteins quantified in at least 1 sample) were as follows: IP1: 924 (570), IP2: 470 (227), IP3: 359 (119).

**Fig 7:**
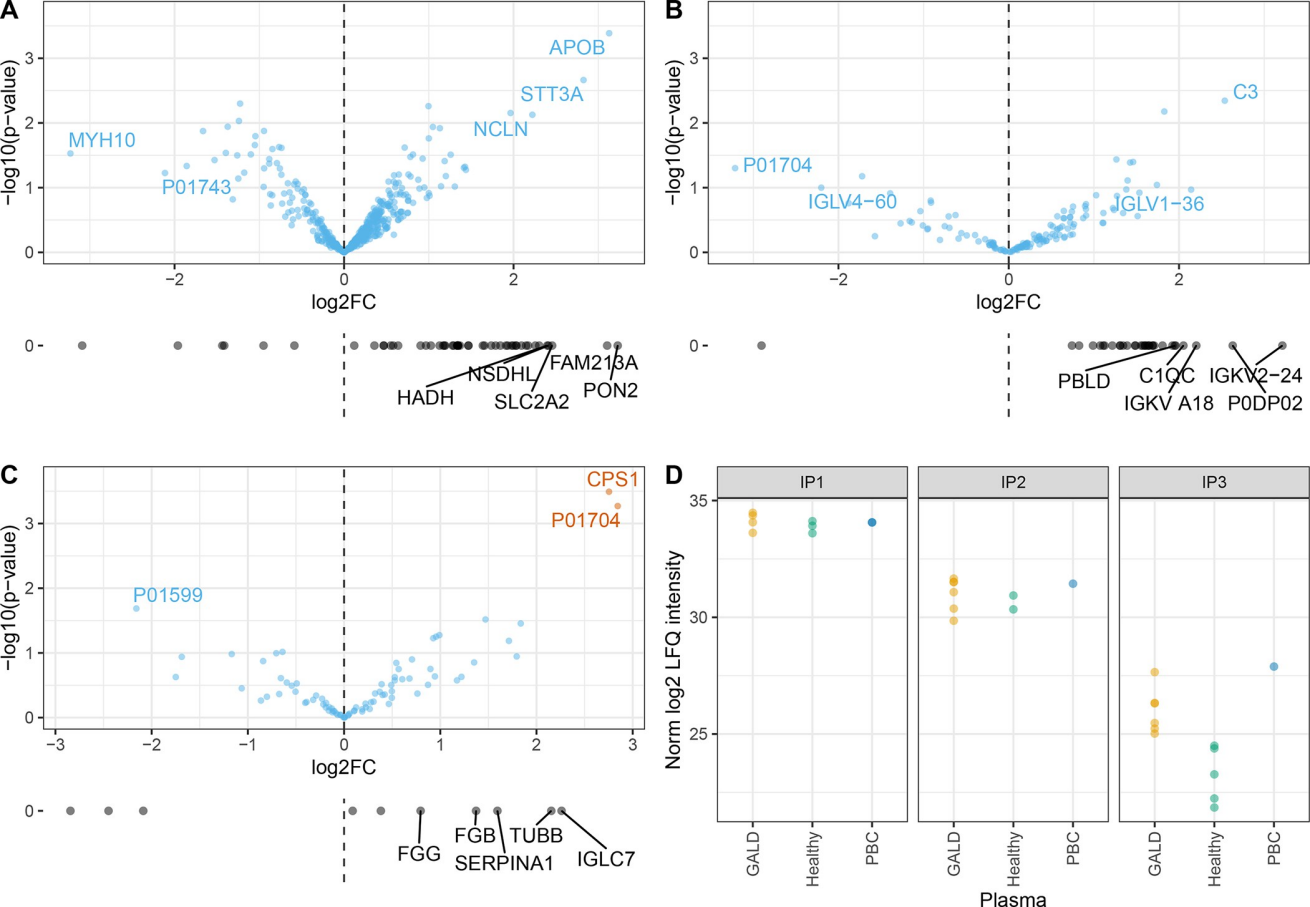
Differential protein abundance in GALD plasma vs healthy controls in fetal liver. A, B, C) Volcano plots depicting differential abundance of proteins in IPs with GALD plasma vs healthy plasma in IP1, IP2 and IP3 respectively. Proteins with Benjamin-Hochberg adjusted p-value (FDR) < 0.05 in orange: Ig lambda chain V-II region TOG (P01704) and Carbamoyl-phosphate synthase 1, mitochondrial (CPS1). Proteins with log_2_ Fold Change > 2 are labeled with gene name or UniProt ID. Plotted below are average log_2_ Fold Change of proteins present in either GALD plasma or healthy controls and in at least 2 samples and the lower 0.1 percentile of normalized LFQ intensities in the respective IP. Top 5 proteins (Fold Change) are labeled. D) Normalized log2 LFQ intensities of CPS1 in 3 conditions GALD, healthy and PBC plasma in 3 IPs. LogFC = log Fold Change. https://github.com/HenriettaHolze/GALD_proteomics/blob/main/scripts/fetal_liver_tissue_analysis.Rmd.

For each liver age (IP1/2 and IP3), no protein group was identified that was absent in all IP samples with healthy plasma and present in all IP samples with GALD plasma based on identified unique peptides. Thus, we then sought proteins that were differentially abundant in IP samples with GALD plasma *versus* IP samples from healthy plasma within each IP. The LIMMA analysis showed only two proteins in IP3 that were significantly differentially abundant in the two groups (FDR < 0.05, Fig 7). One of which was an immunoglobulin and the other one mitochondrial carbamoyl-phosphate synthase (CPS1). CPS1 was also identified and quantified in IP1 and IP2 but not differentially abundant (Fig 7).

Consequently, no plausible candidate antigen was identified in either of the two liver antigen sources: the Huh-7 cell line and the human fetal liver. Nevertheless, we provide the figures with the top fold changes of detected proteins in the different IPs (Fig 7) and results can be found in S5 and S6 Tables with column descriptions in S7 Table.

### Construction from human fetal liver cell mRNA of an expression library in *E*. *coli* and screening with antibody probes

The cDNA expression library in a bacteriophage lambda vector was made from mRNA from human fetal liver cells from several spontaneously aborted fetuses of GA ranging from 22–40. The expression library was screened with antibody probes from two GW. The antibodies were absorbed for anti-*E coli* reactivity.

With an estimated low abundance mRNA of 1:100,000, about 460,000 clones should be screened to find the desired one with a probability of 0.99 using the formula from Maniatis et al. [29]. Even rare mRNA species would be covered with high probability by screening this number of plaques. More than 80% of plaques were white indicating recombinants. Despite screening 1.3 million primary recombinants from an unamplified cDNA library in a lambda expression vector, no reproducibly positive clones were obtained from the cDNA library. 300,000 of the recombinants were screened with plasma eluted from Huh-7 cells. Occasionally a plaque was positive, but none of them could be confirmed by re-plating and re-screening.

### DNA sequencing of exomes and genomes from GW and spouses

Exome sequencing and analysis by Ingenuity variant analysis software was performed on DNA from three unrelated GW as cases and their husbands as controls. Several analytical approaches of the exome data were tested, all requiring the mother to not express a variant present in the father. This included the mother not having a non-essential gene that the father had, or maternal alleles were required to cause a change in the reading frame with a frameshift variant, splice site loss, indel, or a missense mutation. Assuming that all three women were homozygous for the minor allele and all three fathers homozygous for the major allele and the single child was heterozygous resulted in only seven variants (S8 Table). There was no overlap between the six exome candidate proteins and MS-identified proteins. All exome-based candidates had high allele frequencies (S8 Table). Only *POR* and *ADGRE2* were expressed in liver tissue, both fetal and adult. POR is expressed at high levels and considered a membrane protein located to the endoplasmic reticulum, however, only with low levels of expression on the plasma membrane [37, 38]. Our flow cytometry data of Huh-7 showed that some POR protein was accessible for antibody binding on the plasma membrane but also that permeabilization gave more intense staining (results not shown).The frequency of the rs1057868 single nucleotide variant (SNV) (from *POR*) varies among different ethnic populations, but for Caucasians, the allele frequency in the gnomAD database ver 3.1.2 is 0.2886.POR is present in mice and the A503 amino acid is conserved in mice as is the surrounding amino acid sequence of KEPAGEN consistent with experimental findings in mice. However, from the genome sequences of the men 3 were found homozygous and 8 heterozygous and for the women 4 were homozygous (confirming the exome data) and 6 were heterozygous, and two heterozygous children. So, on the above basis the POR variant was ruled out as the causal variant.

The genome sequencing of a larger cohort of 26 GW yielded no targets when sorting for homozygous variants either missense or loss-of-function variants with a minor allelic frequency (MAF) below 10% based on 155K imputed Icelanders (S9 Table). Also, after analysis of data from a trio of one family and an analysis of the mother and child of another family for *de novo* variants (absent from mother), recessive variants (homozygous and compound heterozygous) with MAF<2%, no other candidate gene was found. Only for three genes did the same coordinate or the same amino acid display variants in three or more GW: KAZN (allele frequency 0.0994285) and DDIT4L (allele frequencies 0.0086514 and 0.0907472) and PRKCSH (allele frequencies 0.0362263 and NA).

Ancestry analysis indicated one father was of African ancestry, and one parent family of both mother and father was of South-east-Asian descent, the rest were of Caucasian ancestry.

### Results from HLA analysis

One aspect generally important for immunization risk is the HLA restriction of antibody formation. We did HLA typing of the GW and found no strongly skewed HLA allele frequency (S10 Table). The HLA-C*07:01:01 seemed somewhat rarer among GW. HLA-C has a function in interaction with NK cells many of which are found in uterine wall tissue during pregnancy. The HLA-DRB1*04:04:01, HLA-DRB1*04:05:01, HLA-DRB1*13:02:01 and HLA-DPB1*06:01:01 were somewhat more frequent than expected by comparison to frequencies in a database (http://www.allelefrequencies.net/hla.asp). However, a larger cohort is needed for a conclusive statistical basis.

## Discussion

The central notion that GALD is caused by maternal antibodies is in line with several examples of diseases that are caused by maternal allo-antibodies, e.g.: kidney disease [39], some forms of arthrogryposis multiplex [40], hemolytic disease of the fetus and newborn [41], fetal- and neonatal alloimmune thrombocytopenia (FNAIT) [42], and neonatal alloimmune neutropenia [43].

The identification of the putative fetal liver antigen in GALD would most likely enable development of assays for testing of women at risk of having a baby with GALD, and the development of antenatal risk assessments. However, despite the three-pronged extensive approach in this study, no GALD candidate antigen was identified. Some potentially intriguing candidates from the IP were deemed artifactual, and no candidates emerged from the screening of the expression library. Only a few candidate antigens for GALD were identified from the exome analysis, all with a rather high allele frequency, and of those candidate antigens, only one seemed initially possible and was checked specifically among the genome sequences and was excluded as a candidate based on almost equal occurrence among men and women.

### Immunoprecipitation of liver cell line and human fetal livers

The basis for choosing IP experiments on the Huh-7 hepatocyte cell line was previous results from the FC analysis on fixed Huh-7 liver cells, trypsinized and partially fixed, that showed that IgG from plasma from GW gave a higher signal than control plasma [24]. Most GALD plasmas gave a significantly higher signal than 127 control plasmas from multiparous randomly chosen and anonymized women.

No likely positive candidate antigen was identified by IP in the MS analysis. Complement C1q subunit B was significantly increased and C4 and C3 fragments decreased in IPs with GALD plasma and the Huh-7 cell line. This would be an intriguing finding with direct relevance for GALD; however, in all probability, the finding merely reflects that the patient samples were taken in EDTA tubes whereas citrate tubes were used for the control samples.

The control plasma from a patient with an autoimmune biliary disease did indeed identify some known target antigens of autoantibodies of that disease. In PBC, anti-mitochondrial antibodies that target especially pyruvate dehydrogenase complex, on the inner mitochondrial membrane of biliary endothelial cells [44, 45] served as a positive experimental internal control. The identified positive control antigens were up to 12-fold enriched in the sample with PBC plasma.

The IP could have failed to produce a likely GALD antigen due to the liver cell line not expressing the relevant antigen or antigen(s) despite the FC results showing increased binding of GALD plasma to Huh-7. The antigen might have been degraded or denatured during the procedures, e.g., it might be sensitive to freezing or detergents, or the antibodies did not bind the antigen with the necessary strength under the conditions used. Finally, but perhaps more unlikely, the antigen was not identified because it was not listed in the reference database used for MS identification.

The putative GALD antigen (or a subcomponent) might be produced outside the liver and then transported to the liver; still, expression in the liver is the more attractive notion. Nevertheless, it was surprising that no antigen was identified as the Huh-7 cell line reacted with the GALD plasma antibodies as shown by FC of Huh-7, where a convincing and reproducible signal was found.

No protein antigen could be identified that was absent in controls and present in samples of frozen human fetal liver samples from late abortions as antigen source using GALD plasma. The only plausible protein candidate enriched in samples with GALD plasma and fetal liver material was CPS1, known to be located to nucleoli. However, CPS1 was not enriched in GALD samples in the other IPs and thus no plausible candidate antigen could be identified.

### Expression library

Screening lambda expression libraries can be a powerful method [46]. The screening of 1.3 million clones of the expression library constructed from fetal liver mRNA, however, yielded no candidate antigens. Despite that the screening was performed with plasma either absorbed for anti-*E coli* reactivity or antibody eluted from fixed Huh-7 cells, no consistently positive clones were isolated. The screenings performed with eluted antibodies gave a very low background. Some of the reasons for not finding any positive clones could be that the target antigen requires posttranslational processing in eukaryotic cells for antibodies to bind or consists of a hetero-multimeric protein where the target epitopes are made up from different proteins or that the correct folding is not possible in an *E*. *coli* environment. If the GALD antigen is only expressed at a very low level and in precursor cells, perhaps many more plaques should have been screened. Perhaps the GALD antigen is produced in another tissue than liver and transported to the liver where it is displayed on the fetal liver surface. This possibility would also preclude identification from a fetal liver cDNA expression library.

### Exome/Genome analysis

In a biallelic system, a rare disease situation with maternal-fetal allotype incompatibility can arise either if the woman is homozygous for a very rare allele and the man (most likely) is homozygous for the frequent contrasting allele or, alternatively, if the woman is homozygous for a very frequent allele with the man being homozygous or more likely heterozygous for the contrasting rare allele (S1 Fig).

This genetic basis is not sufficient for a disease of maternal-fetal alloimmune incompatibility, merely a prerequisite. The following line of arguments gives credence to the opinion that the putative GALD antigen, if it is indeed a single alloantigen, must have its genetic basis in a rare allele in the women, not a frequent one. This argument is important for the bioinformatic analysis of the genetic sequencing data and rests on epidemiological information. The GW, who have babies with other men, continue to have GALD babies with unaltered recurrence rates. If the GW were homozygous for a high frequency allele, the vast majority of men would also have the high frequency allele—assuming no gender bias—and another random man would then be very unlikely to have the rare allele expressing the antithetical antigen to be targeted by maternal antibodies when expressed in the fetus. Furthermore, a random man carrying the rare allele would most likely be heterozygous and therefore only about 50% of the babies would be expected to be affected. However, if the GW are homozygous for a very rare allele, a random man would most likely be homozygous for the frequent contrasting allele, and thus another, unrelated man would again be most likely to be homozygous for the frequent allele and contribute the same high frequency allele to all his babies. The inescapable conclusion from these considerations is that only a woman, who is homozygous for a rare allele would explain the rarity of the disease as well as the epidemiological observations, assuming that the causal antigen is an alloantigen in a biallelic system. A recurrence rate of 95% [15] would also directly reflect the frequency of the putative minor allele of 0.05 in a biallelic situation with 100% penetrance. This figure is important for choices pertaining to the bioinformatics analysis. The possible requirement for the presence of other factors to elicit GALD, though not antibody production, might somewhat invalidate these simple deliberations.

By exome sequencing, we identified several SNVs resulting in amino acid change that were present in a homozygous form in the three mothers but not in the fathers and in the heterozygous constellation in the single child included. The high frequency of the allotype candidates TMEM40, TGM4, KRT75 found by exome analysis would make them unlikely candidates. Even though the haplotype frequency of the combined KRT6B variants is not known, this haplotype is not rare judging from the results from other exomes in other projects (results not shown). Furthermore, the known expression pattern of these molecules also makes them unlikely as GALD antigen candidates. POR and ADGRE2 have an allele frequency of almost 30% which is still rather high considering the recurrence rate of the disease. Also, the expression pattern of ADGRE2 as taken from a database makes this candidate less likely although we note a high mRNA expression in Kupffer cells (https://www.proteinatlas.org/ENSG00000127507-ADGRE2). POR is an important molecule for detoxification processes in the liver, and it is a membrane protein expressed mostly in the endoplasmic reticulum but with low levels of expression on the plasma membrane [37]. Also, the C-terminal part of POR carrying the A503V amino acid substitution is predicted to be in the cytoplasm. To conclusively rule out POR, additional samples from mothers and fathers of one or more children diagnosed with GALD were examined by genome sequencing.

Of the genetic variants identified in the exome study, the SNV in POR seemed to constitute the best possible target of maternal alloantibodies due to the expression in liver cells. The frequency of the minor allele though is so high that this candidate was given thorough, special consideration. In a biallelic system, a minor allele frequency of about 30% would result in a recurrence rate of about 70%, at the population level, which is a fair bit away from the observed recurrence rate of at least 90% [6, 15]. This assumes that only a single antigen in a biallelic locus is involved.

However, even though POR could be an interesting target, the conclusion from these deliberations was that we did not identify a GALD target antigen by the exome analysis.

The exome analyses were extended with 26 genome analyses of GW. No new GALD target antigen candidates with allele frequencies of less than 10 percent were found. We find that none of the homozygous variants are credible targets of maternal antibodies. The lack of a credible alloantigen would mean that the daughters of a GW would be unlikely to give birth to a GALD baby, at least based on simple Mendelian inheritance.

### HLA considerations

It is generally agreed that the immunization risk and production of specific antibodies against a protein antigen is modulated by the HLA presentation of peptides from the antigen [47]. There are many examples that a specific HLA type is conducive to the development of a given antibody specificity [48–50]. We found no striking over- or underrepresentation of specific HLA alleles in GW. This does not rule out the hypothesis that GALD is mediated by maternally produced antibodies reacting against a specific fetal liver protein antigen. The HLA-A*03:01 allele is not rare and about 18% of Americans carry it. The HLA-C*07 allele is also very frequent, and more frequent among Caucasians than other ethnicities, and the class II antigen DPA*01:03:01 common to several GW is also frequent. The lack of a strong HLA association would imply that the daughter of a GW would be unlikely herself to give birth to a baby with GALD. Testing HLA genes in a larger cohort, however, might better answer whether a specific HLA type would be associated with GALD.

### Alloantigen or fetal expression only, or some other etiology?

GALD occurs in families, but there is conflicting epidemiological evidence whether the disease is also inheritable, but most favored as non-heritable [6, 13]. In some families, autosomal recessive inheritance has been suggested [23]. Some of the reported epidemiological data are: The recurrence rate of lethal disease after the index case is as high as 95% [15]. Many women have had several normal babies before the index case. There are several instances of women having affected offspring with different fathers [51, 52]. No sisters of affected women have ever been reported to have a baby with GALD [1]. Due to the rarity of the disease, it is hard to draw any firm conclusions from this epidemiological observation except that dominant Mendelian inheritance is unlikely. The fecundity among western women is low (https://data.worldbank.org/indicator/SP.DYN.TFRT.IN) and about 15% of women aged 45 in US have not had children (https://www.statista.com/statistics/241535/percentage-of-childless-women-in-the-us-by-age/). This means that it would be rare that two sisters both had a GALD child; thus, the absence of reports of sisters of GALD mothers weighs against an alloantigen origin of the disease but is not conclusive. Now our inability to identify a candidate antigen from the exome and genome analyses speaks against the hypothesis that the putative GALD antigen is an alloantigen. We did not interrogate experimentally the possibility of a viral etiology and this possibility seems remote [53, 54]. A hypothesis of the putative GALD antigen being expressed only during fetal life is in accordance with most epidemiological data and seems plausible.

### Why was the GALD antigen not identified?

We used three different comprehensive technological approaches attempting to identify the putative GALD antigen targeted by maternal antibodies. Each method has its advantages and biases but despite these comprehensive attempts, we were unable to identify an antigen as a target of maternal antibodies from women who had given birth to one or more children with GALD.

The IP is a complex procedure and requires the presence of the target antigen in a native configuration in sufficient amount to be precipitated and identified. Furthermore, the antigen-antibody affinity must be sufficient under the experimental IP conditions used. The library screened in *E*. *coli* would have the limitations described above. The genetics approach would not be useful in the case of an antigen that is only expressed during fetal life. Even though objections could be raised against technical aspects of each approach, the combined results left us to favor one or more exclusively fetally expressed antigen(s) as the target of maternal antibodies explaining the etiology of GALD.

The iron overload in GALD has been explained as lack of hepcidin production from the affected liver [13] but this explanation has been challenged by others [55]. So, the relation to iron metabolism is not entirely clear, the increase in iron deposition in several organs cannot be explained currently but may be a result of liver failure in the fetus. C5b9 histological staining of liver samples has been used to classify the newborn liver disease into GALD or non-GALD disease [14, 56]. The value of C5b9 staining of liver samples in GALD, however, has been questioned [20], and novel more specific diagnostic approaches will be useful.

The speculation that an unknown virus lays at the root of an enigmatic disease is often the last refuge of a doctor when confronted with a disease of unproven etiology. We felt, however, compelled to ponder this possibility as already suggested by others at a time before viral hepatitis etiology was well described [57, 58]. Still, other speculative possibilities include but are not limited to catalytic antibodies or agonistic antibodies both with rapid dissociation would make identification difficult using the simple IP protocol. It has been reported that the placenta showed maturation deficits at birth of a GALD baby [59]. The somewhat remote possibility of an entirely different etiology or pathogenesis cannot be completely ruled out presently.

The prevailing hypothesis that explains the cause of GALD as production in an immunized woman of antibodies reactive with an antigen located to the liver of the fetus seems very plausible. The antibodies would pass over the placenta into the fetus and specifically react with an antigen expressed on fetal liver cells and this reaction would start an immunological destruction of the fetal hepatocytes and spare the biliary ducts. This hypothesis fits well with the epidemiology of the disease, some experimental results in mice, and the clinical observation of a beneficial effect of IVIG treatment for attenuation or full prevention of clinical disease in babies born from a woman who has previously given birth to a baby with GALD [60]. There is a striking effect of IVIG given in early pregnancy in preventing GALD in pregnancies where about 90–95% recurrence would be expected if left untreated. The IVIG effect to prevent GALD recurrence appears to be more efficient than when IVIG is used in, e.g., women with alloimmunization against fetal blood group antigens or the platelet antigen, HPA1. In these latter cases, alloantibodies are known to be the cause of the immunological destruction of the fetal red blood cells or platelets, yet IVIG treatment cannot prevent disease, only slightly mitigate it. This possible discrepancy in the effect of IVIG might indicate that some other unidentified biological processes are at play in GALD. The lack of a strong HLA association with GALD could help keep an open mind as to the etiology.

To call into question the idea of a single GALD antigen, expressed in fetal liver, is premature, but if continued attempts in line with this hypothesis to identify the elusive GALD antigen fail, other possibilities of pathogenesis should be considered. Other possibilities as for instance an exogenous causative agent, e.g., a virus would be missed in all three approaches. Effects of disease located to the placenta/uterus in the form of, e.g., a chronic infection under normal conditions pauci-symptomatic or completely subclinical is perhaps a remote possibility. It may be wise not to take for granted that all women in this study have the same disease with the same molecular causality, rather several diseases with a closely matching clinical presentation is a possibility.

In conclusion, we were not able to identify the target antigen of the GALD disease despite a massive effort and thus, we have been unable to develop new and urgently needed diagnostic laboratory tools for improved diagnosis of GALD and advance the understanding of the disease. Due to the negative findings from the extensive genetic analyses, it seems unlikely based on our results, that the GALD antigen is an alloantigen, and we favor the hypothesis that the antigen is exclusively expressed during fetal life in all pregnancies.

## Supporting information

S1 Fig Maternal-fetal genetic constitution in a biallelic system. The probability of maternal homozygosity with one allele and a fetus with the contrasting allele encoding the antithetic antigen in a biallelic system in a population is shown. The probability was calculated as p^2^_*_(1-p) = p^2^-p^3^, where p is the frequency of the maternal allele. The complimentary distribution is defined by (p-1)^2^p and not shown.
(TIF)

S1 Table Overview of immunoprecipitation experiments with fetal liver tissue. (DOCX)

S2 Table Experimental design of immunoprecipitation from Huh7. (XLSX)

S3 Table Experimental design of immunoprecipitation from fetal liver. (XLSX)

S4 Table PBC targets used for control in IP experiments. A control plasma from a patient with autoimmune liver disease efficiently precipitated components of mitochondrial pyruvate dehydrogenase complex, known as autoantigens in primary biliary cirrhosis.
(DOCX)

S5 Table MS results from immunoprecipitated antigens from Huh7. No obvious GALD antigen candidate was identified.
(XLSX)

S6 Table MS results from immunoprecipitated antigens from fetal liver. The MS results from immunoprecipitation from human fetal liver showed no obvious candidate.
(XLSX)

S7 Table Column descriptions. (XLSX)

S8 Table Exome based candidates satisfying selected criteria. Variants found to satisfy the criteria of the women being homozygous with a minor allele and the men being homozygous with the major allele. The six potential candidate variants identified from exome/genome sequencing are listed with coordinate, amino acid substitution, rs-number, the liver expression level, and subcellular location (https://www.proteinatlas.org/, Version: **21.0** Atlas updated: 2021-11-18).
(DOCX)

S9 Table Genome sequencing results. Showing homozygous variants found in 26 GW with an allele frequency of less than 10%. The instances where 3 or more women were homozygous for variants in the same gene is marked in bold. Only for three genes did the same coordinate or the same amino acid have variants in 3 or more GW: KAZN (allele frequency 0,0994285) and DDIT4L (allele frequencies 0,0086514 and 0,0907472) and PRKCSH (allele frequencies 0,0362263 and NA).
(XLSX)

S10 Table HLA typing results from 16 GW. No single HLA haplotype is common for the GW. The HLA region was DNA sequenced on a PacBio instrument to ascertain the haplotype. No single HLA type was found with a frequency significantly different from published frequencies with Europeans as reference.
(DOCX)

S1 File (TXT)

S2 File (ZIP)

## Abbreviations

aa: amino acid
EDTA: Ethylenediaminetetraacetic acid
FBS: fetal bovine serum
FC: flow cytometry
FDR: false discovery rate
GA: gestational age
GALD: Gestational alloimmune liver disease
GW: woman who has given birth to a child diagnosed with GALD
HLA: human leukocyte antigen
IVIG: intravenous immunoglobulin
IP: immunoprecipitation
LB: Luria Broth
LFQ: label free quantification
LIMMA: Linear Models For Microarray Data
MAF: minor allelic frequency
mRNA: messenger RNA
MS: Mass spectrometry
NA: not applicable
PBC: primary biliary cirrhosis
PCA: Principal component analysis
POR: Cytochrome p450 oxidoreductase
ppm: parts per million
RT: room temperature
SNV: single nucleotide variation
