# Supplementary figures and images for: Hunting for the elusive target antigen in gestational alloimmune liver disease (GALD)
Source: PLoS One. 2023 Oct 20;18(10):e0286432. doi: 10.1371/journal.pone.0286432 (PMC10588877; doi:10.1371/journal.pone.0286432)

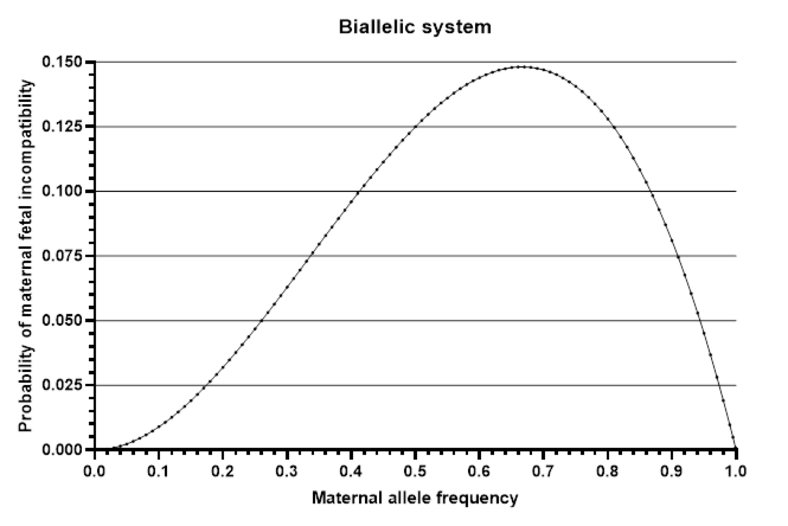

Supplement: S1 Fig — The probability of maternal homozygosity with one allele and a fetus with the contrasting allele encoding the antithetic antigen in a biallelic system in a population is shown. The probability was calculated as p2*(1-p) = p2-p3, where p is the frequency of the maternal allele. The complimentary distribution is defined by (p-1)2p and not shown. (TIF) [file pone.0286432.s001.tif]
