# Supplementary material for: Hunting for the elusive target antigen in gestational alloimmune liver disease (GALD)
Source: PLoS One. 2023 Oct 20;18(10):e0286432. doi: 10.1371/journal.pone.0286432 (PMC10588877; doi:10.1371/journal.pone.0286432)
Supplement: S1 Table — (DOCX) [file pone.0286432.s002.docx]

**S1 Table** **Overview of immunoprecipitation experiments with fetal liver material.**

|  | **IP1** | **IP2** | **IP3** |
| --- | --- | --- | --- |
| Preparation | 1) Homogenized + sonicated  2) Homogenized  3) pre-incubated | Homogenized + sonicated | Homogenized + sonicated and liver lysate pre-cleared with AB plasma |
| Fetal liver age | GA19 | GA19 | GA 21 |
| Samples with healthy plasma | 3 | 2 | 5 |
| Controls w/o plasma | 1 | 0 | 1 |
| Samples with GALD plasma | 6 (2 patients) | 6 + 1 under IVIG treatment | 6 + 1 under IVIG treatment |
| Samples with PBC plasma | 3 (1 patient) | 1 | 1 |
