# Supplementary material for: Hunting for the elusive target antigen in gestational alloimmune liver disease (GALD)
Source: PLoS One. 2023 Oct 20;18(10):e0286432. doi: 10.1371/journal.pone.0286432 (PMC10588877; doi:10.1371/journal.pone.0286432)
Supplement: S4 Table — A control plasma from a patient with autoimmune liver disease efficiently precipitated components of mitochondrial pyruvate dehydrogenase complex, known as autoantigens in primary biliary cirrhosis. (DOCX) [file pone.0286432.s005.docx]

S4 Table. PBC targets

| Antigen targets in PBC |  |
| --- | --- |
| P10515 | Dihydrolipoyllysine-residue acetyltransferase component of pyruvate dehydrogenase complex, mitochondrial |
| P09622 | Dihydrolipoyl dehydrogenase, mitochondrial |
| P11177 | Pyruvate dehydrogenase E1 component subunit beta, mitochondrial |
| P08559 | Pyruvate dehydrogenase E1 component subunit alpha, somatic form, mitochondrial |
| O00330 | Pyruvate dehydrogenase protein X component, mitochondrial |
| O60232 | Sjoegren syndrome/scleroderma autoantigen |
