# Supplementary material for: Hunting for the elusive target antigen in gestational alloimmune liver disease (GALD)
Source: PLoS One. 2023 Oct 20;18(10):e0286432. doi: 10.1371/journal.pone.0286432 (PMC10588877; doi:10.1371/journal.pone.0286432)
Supplement: S8 Table — Variants found to satisfy the criteria of the women being homozygous with a minor allele and the men being homozygous with the major allele. The six potential candidate variants identified from exome/genome sequencing are listed with coordinate, amino acid substitution, rs-number, the liver expression level, and subcellular location (https://www.proteinatlas.org/, Version: 21.0 Atlas updated: 2021-11-18). (DOCX) [file pone.0286432.s009.docx]

S8 Table. Exome based candidates satisfying selected criteria

| Gene symbol | Name | Amino acid | rs number | Maternal allele frequency ¤ | Normal liver expression | Main subcellular localization |
| --- | --- | --- | --- | --- | --- | --- |
| TMEM40 | transmembrane protein 40 | p.P12R | rs7641959 | 0.6305 | no | membrane |
| TGM4 | transglutaminase 4 | p.E313K | rs1995641 | 0.4218 | no | intracellular |
| POR | cytochrome p450 oxidoreductase | p.A503V | rs1057868 | 0.2858 | High levels | Intracellular membrane |
| KRT75 | keratin 75 | p.R91G | rs298109 | 0.5843 | no | intracellular |
| KRT6B | keratin 6B | p.I365V | rs437014 | 0.4801 | no | intracellular |
| KRT6B | keratin 6B | p.N21S | rs428894 | 0.4984 | no | intracellular |
| ADGRE2 | adhesion G protein-coupled receptor E2 | p.L160V | rs12976472 | 0.4928 | no or very low levels | membrane |

¤ allele frequencies were taken from gnomAD v 3.1.1 for Europeans (non-Finnish)
