# Supplementary material for: Hunting for the elusive target antigen in gestational alloimmune liver disease (GALD)
Source: PLoS One. 2023 Oct 20;18(10):e0286432. doi: 10.1371/journal.pone.0286432 (PMC10588877; doi:10.1371/journal.pone.0286432)
Supplement: S10 Table — No single HLA haplotype is common for the GW. The HLA region was DNA sequenced on a PacBio instrument to ascertain the haplotype. No single HLA type was found with a frequency significantly different from published frequencies with Europeans as reference. (DOCX) [file pone.0286432.s011.docx]

|  | **Typing results with NMDP code** | | | | | | | | | | | | | | | | | | | | | | |
| --- | --- | --- | --- | --- | --- | --- | --- | --- | --- | --- | --- | --- | --- | --- | --- | --- | --- | --- | --- | --- | --- | --- | --- |
| sample | **A1** | **A2** | **B1** | **B2** | **C1** | **C2** | **DRB11** | **DRB12** | **DRB31** | **DRB32** | **DRB41** | **DRB42** | **DRB51** | **DRB52** | **DQB11** | **DQB12** | **DQA11** | **DQA12** | **DPB11** | **DPB12** | **DPA11** | **DPA12** |  |
| 1 | 01:01:01:01 | 24:02:01 | 07:02:01 | 57:01:01 | 02:02:02 | 06:02:01 | 01:01:01 | 07:01:01 |  |  | 01:03:01:02N |  |  |  | 03:03:02:01 | 05:01:01:03 | 01:01:01 | 02:01:01 | 03:01:01 | 04:01:01 | 01:03:01 | 01:03:01 |  |
| 2 | 11:01:01 | 31:01:02:01 | 08:01:01 | 37:01:01:01 | 06:02:01 | 07:02:01 | 03:01:01:03 | 13:02:01 | 02:02:01 | 03:01:01 |  |  |  |  | 02:01:01 | 06:09:01:01 | 01:02:01 | 05:01:01 | 104:01:01 | 04:01:01 | 01:03:01 | 01:03:01 |  |
| 3 | 01:01:01:01 | 11:01:01 | 38:01:01:01 | 44:02:01 | 05:01:01 | 06:02:01 | 04:03:01:01 | 13:02:01 | 03:01:01:XX |  | 01:03:01 |  |  |  | 03:04:01 | 06:09:01:01 | 01:02:01 | 03:01:01 | 04:01:01 | 10:01:01:01 | 01:03:01 | 02:01:01 |  |
| 4 | 02:06:01:01 | 26:01:01:01 | 38:01:01:01 | 48:01:01 | 08:03:01 | 12:03:01 | 04:02:01 | 04:03:01:01 |  |  | 01:03:01 | 01:03:01 |  |  | 03:04:01 | 03:ADAJH | 03:01:01 | 03:01:01 | 02:01:02 | 04:01:01 | 01:03:01 | 01:03:01 |  |
| 5 | 03:01:01:01 | 03:01:01:01 | 07:02:01 | 44:03:01 | 07:02:01 | 16:01:01 | 04:04:01:XX | 15:01:01 |  |  | 01:03:01 |  | 01:01:01:01 |  | 03:ADAJH | 06:02:01:01 | 01:02:01 | 03:01:01 | 03:01:01 | 04:01:01 | 01:03:01 | 01:03:01 |  |
| 6 | 02:01:01 | 03:01:01:01 | 07:02:01 | 07:02:01 | 07:02:01 | 07:02:01 | 04:04:01:XX | 15:01:01 |  |  | 01:03:01 |  | 01:01:01:01 |  | 03:ADAJH | 06:02:01:01 | 01:02:01 | 03:01:01 | 04:01:01 | 06:01:01 | 01:03:01 | 01:03:01 |  |
| 7 | 03:01:01:01 | 03:01:01:01 | 08:01:01 | 15:01:01 | 03:04:01:XX | 07:01:01 | 03:01:01 | 11:01:01 | 01:01:02 | 02:02:01:02 |  |  |  |  | 02:01:01 | 03:BKHVR | 05:01:01:02 | 05:EF | 01:01:01 | 04:01:01 | 01:03:01 | 02:01:02:02 |  |
| 8 | 01:01:01:01 | 02:01:01 | 07:02:01 | 44:02:01 | 06:02:01 | 07:02:01 | 04:04:01 | 15:01:01 |  |  | 01:03:01 |  | 01:01:01:01 |  | 03:ADAJH | 06:02:01:01 | 01:02:01 | 03:01:01 | 02:01:02 | 04:01:01 | 01:03:01 | 01:03:01 |  |
| 9 | 03:01:01:01 | 32:01:01 | 27:05:02 | 57:01:01 | 02:02:02 | 06:02:01 | 04:01:01 | 13:01:01 | 02:02:01:02 |  | 01:03:01 |  |  |  | 03:BGCWT | 06:03:01 | 01:03:01 | 03:03:01 | 03:01:01 | 16:01:01:01 | 01:03:01 | 01:03:01 |  |
| 10 | 01:01:01:01 | 33:03:01 | 52:01:01 | 58:01:01 | 03:02:02 | 12:02:02 | 03:01:01:03 | 15:BFBXD | 02:02:01:01 |  |  |  | 01:02 |  | 02:01:01 | 06:01 | 01:03:01 | 05:01:01 | 02:01:02 | 23:01:01:01 | 01:03:01 | 01:03:01 |  |
| 11 | 23:01:01:01 | 24:02:01 | 07:02:01 | 44:03:01 | 04:01:01 | 07:02:01 | 04:04:01:XX | 15:01:01 |  |  | 01:03:01 |  | 01:01:01:01 |  | 03:ADAJH | 06:02:01:01 | 01:02:01 | 03:01:01 | 04:01:01 | 20:01:01 | 01:03:01 | 01:03:01 |  |
| 12 | 02:01:01 | 02:01:01:29 | 44:02:01 | 51:01:01 | 05:01:01 | 15:02:01:01 | 04:04:01:XX | 08:01:01 |  |  | 01:03:02 |  |  |  | 03:ADAJH | 04:02:01 | 03:01:01 | 04:01:01 | 03:01:01 | 04:01:01 | 01:03:01 | 01:03:01 |  |
| 13 | 02:01:01 | 03:01:01:01 | 14:02:01:01 | 57:01:01 | 06:02:01 | 08:02:01:01 | 04:04:01:XX | 15:01:01 |  |  | 01:03:01 |  | 01:01:01:01 |  | 03:ADAJH | 06:02:01:01 | 01:02:01 | 03:01:01 | 04:01:01 | 06:01:01 | 01:03:01 | 01:03:01 |  |
| 14 | 02:01:01 | 68:01:02:02 | 44:02:01 | 44:02:01:03 | 05:01:01 | 07:04:01 | 01:03:01 | 04:01:01 |  |  | 01:03:01 |  |  |  | 03:BGCWT | 05:01:01:03 | 01:01:01 | 03:03:01 | 02:01:02 | 06:01:01 | 01:03:01 | 01:03:01 |  |
| 15 | 01:01:01:01 | 03:01:01:01 | 08:01:01 | 44:02:01 | 05:01:01 | 07:01:01 | 03:01:01 | 04:01:01 | 01:01:02:02 |  | 01:03:01 |  |  |  | 02:01:01 | 03:BGCWT | 03:03:01 | 05:01:01:02 | 01:01:01 | 04:01:01 | 01:03:01 | 02:01:02:02 |  |
| 16 | 02:01:01 | 03:01:01:01 | 07:02:01 | 40:01:02 | 03:04:01 | 07:02:01 | 04:05:01 | 13:02:01 | 03:01:01 |  | 01:03:01 |  |  |  | 03:ADAJH | 06:04:01 | 01:02:01 | 03:03:01 | 03:01:01 | 124:01:02 | 01:03:01 | 01:03:01 |  |

S10 Table. HLA types from 16 GALD women
